# Typical investigational medicinal products follow relatively uniform regulations in 10 European Clinical Research Infrastructures Network (ECRIN) countries

**DOI:** 10.1186/1745-6215-13-27

**Published:** 2012-03-27

**Authors:** Christian Gluud, Christine Kubiak, Kate Whitfield, Jane Byrne, Karl-Heinz Huemer, Steffen Thirstrup, Christian Libersa, Béatrice Barraud, Xina Grählert, Gabriele Dreier, Sebastian Geismann, Wolfgang Kuchinke, Zsuza Temesvari, Gyorgy Blasko, Gabriella Kardos, Timothy O'Brien, Margaret Cooney, Siobhan Gaynor, Arrigo Schieppati, Fernando de Andres, Nuria Sanz, German Kreis, Charlotte Asker-Hagelberg, Hanna Johansson, Sue Bourne, Adeeba Asghar, Jean-Marc Husson, Jacques Demotes-Mainard

**Affiliations:** 1 Copenhagen Trial Unit (CTU), Centre for Clinical Intervention Research, and the Danish Clinical Research Infrastructures Network (DCRIN), Rigshospitalet, Copenhagen University Hospital, Copenhagen, Denmark; 2 Institut Thématique Santé Publique, Institut National de la Santé et de la Recherche Médicale (Inserm), Paris, France; 3 Education and Research Centre, Wythenshawe Hospital, Manchester, UK; 4 Medical University of Vienna (ATCRIN), Vienna, Austria; 5 Danish Medicines Agency, Copenhagen, Denmark; 6 Clinical Investigation Centre CH&U-INSERM 9301, Lille, France; 7 KKS, Medizinische Fakultät Carl Gustav Carus, Dresden, Germany; 8 Studienzentrum-Clinical Trials Unit, University Medical Centre Freiburg, Freiburg, Germany; 9 KKS-Duesseldorf (KKSD), Heinrich-Heine University, Duesseldorf, Germany; 10 Ministry of Health Social and Family Affairs, Hungarian ECRIN Committee, Medical Research Council (HECRIN), Budapest, Hungary; 11 National University of Ireland, Galway, Ireland; 12 Irish Clinical Research Infrastructures Network (ICRIN), Dublin; 13 Istituto di Ricerche Farmacologiche Mario Negri (IRFMN), Milano, Italy; 14 Departemento de Farmacologia (Medicina), Universidad Complutense, Madrid, Spain; 15 Hospital Clinic i Provincial de Barcelona (SCReN), Barcelona, Spain; 16 Karolinska Trial Alliance, Stockholm, Sweden; 17 UK Clinical Research Collaboration, London, UK; 18 NIHR Clinical Research Network Coordination Centre, UK; 19 European Forum for Good Clinical Practice, Brussels, Belgium

## Abstract

**Background:**

In order to facilitate multinational clinical research, regulatory requirements need to become international and harmonised. The EU introduced the Directive 2001/20/EC in 2004, regulating investigational medicinal products in Europe.

**Methods:**

We conducted a survey in order to identify the national regulatory requirements for major categories of clinical research in ten European Clinical Research Infrastructures Network (ECRIN) countries-Austria, Denmark, France, Germany, Hungary, Ireland, Italy, Spain, Sweden, and United Kingdom-covering approximately 70% of the EU population. Here we describe the results for regulatory requirements for typical investigational medicinal products, in the ten countries.

**Results:**

Our results show that the ten countries have fairly harmonised definitions of typical investigational medicinal products. Clinical trials assessing typical investigational medicinal products require authorisation from a national competent authority in each of the countries surveyed. The opinion of the competent authorities is communicated to the trial sponsor within the same timelines, i.e., no more than 60 days, in all ten countries. The authority to which the application has to be sent to in the different countries is not fully harmonised.

**Conclusion:**

The Directive 2001/20/EC defined the term 'investigational medicinal product' and all regulatory requirements described therein are applicable to investigational medicinal products. Our survey showed, however, that those requirements had been adopted in ten European countries, not for investigational medicinal products overall, but rather a narrower category which we term 'typical' investigational medicinal products. The result is partial EU harmonisation of requirements and a relatively navigable landscape for the sponsor regarding typical investigational medicinal products.

## Background

Clinical trials are studies conducted with human trial participants with the intention to increase knowledge about how well a diagnostic test or therapeutic intervention works in a particular population. Randomised clinical trials are the most stringent type of clinical research to determine efficacy and safety, only surpassed by systematic reviews of such trials [1-4]. However, most clinical trials are conducted using inadequate methodologies and involve too few participants [5-12]. This raises the risks of systematic errors and random errors. The tasks for clinical research are further challenged by the necessity to study the impact of different genetic subgroups on the intervention effect [13,14]. The summed consequences are that we need extensive research collaboration to secure adequate methodologies and sufficiently large participant numbers [15,16]. One way to attack these problems is to conduct multinational clinical trials.

Multinational clinical trials require the sponsor to navigate multiple legislative systems and regulatory environments. The objective of the Directive 2001/20/EC was to harmonise clinical trial regulations within the EU member states for investigational medicinal products [17]. The opposite effect has been alluded to in a number of articles and reports, citing evidence that the implementation of the Directive 2001/20/EC resulted in divergences at the national level and increased complexity in conducting both national as well as multinational clinical trials [18-26].

Knowledge of the different national regulatory requirements is an essential prerequisite for conducting multinational clinical research, and whereas the larger commercial companies have such provisions, the academic sector and small and medium sized companies are largely without this body of knowledge. The European Clinical Research Infrastructures Network (ECRIN) aims at integrating clinical research in Europe through connecting national networks of clinical research centres and clinical trial units. ECRIN helps investigators and sponsors navigate the minefield of regulatory issues which differ across boarders and provide information, consultation and access to services necessary to conduct multi-centre clinical studies for the benefit of patient and citizens [15,16].

ECRIN's Working Group on regulatory requirements and interaction with competent authorities had the task to describe such regulatory requirements and delineate how to interact with competent authorities in the countries involved in ECRIN, covering approximately 70% of the EU population [27]. We therefore conducted a survey to identify the national requirements for all categories of clinical research defined and agreed upon by the Working Group [27]. The present article describes the results for regulatory requirements during the approval phase of clinical trials on typical investigational medicinal products, in the ten ECRIN countries.

## Methods

In order to analyse the current situation regarding the regulatory environment during the approval phase of clinical research in Europe, we designed the survey as described in a previous publication [27]. The full survey questionnaire is available as online supplementary material http://www.ecrin.org. For the purpose of the survey we identified seven common definitions for categories of clinical research, each split into sub-categories [27]. One of the sub-categories was clinical research assessing typical investigational medicinal products (Table 1). Typical investigational medicinal products comprise in general chemically synthesised drugs, constituting the active substance within an investigational medicinal product. Such typical investigational medicinal products are used in phase I to IV clinical trials independently of the question, if they have been authorised before for the investigative or for any other use. Less typical investigational medicinal products, mainly biopharmaceutical investigational medicinal products and a variety of other less typical investigational medicinal products (Table 1) are also assessed in phase I to IV clinical trials, but are not dealt with in the results section of the present article. Our responses to the survey questionnaire on both typical investigational medicinal products and less typical investigational medicinal products have been reported to the European Commission in 2008 and are available at http://www.ecrin.org/index.php?id=101[28].

**Table 1:** Definition of clinical trials on typical investigational medicinal products as well as less typical investigational medicinal products.

Category	Includes
Clinical trials on typical investigational medicinal products	Phase I to IV clinical trials on medicinal products, that is any substance or combination of substances presented for treating or preventing disease in human beings. Any substance or combination of substances which may be administered to human beings with a view to making a medical diagnosis or to restoring, correcting or modifying physiological functions in human beings is likewise considered a medicinal product.

Clinical trials on biopharmaceutical investigational medicinal products or other less typical investigational medicinal products	Phase I to IV clinical trials on
	- Biological medicinal products, i.e., proteins preceded either from biological material by standardised purification techniques or by recombinant DNA technologies or hybridoma or monoclonal antibody methods.
	- Vaccines.
	- Advanced therapy medicinal products, consisting of
	- gene therapy medicinal products (human or xenogeneic);
	- somatic cell therapy medicinal products (human or xenogeneic);
	- tissue engineered product, i.e., a product that contains or consists of engineered cells or tissues (human or xenogeneic).
	- Plasma-derived medicinal products.
	- Blood products.
	- Radio-pharmaceuticals and precursors.
	- Homeopathic medicinal products.
	- Herbal medicinal products.

The ECRIN Working Group on regulatory requirements and interaction with competent authorities was composed of two chairpersons and at least two representatives from each national network from Austria, Denmark, France, Germany, Hungary, Ireland, Italy, Spain, Sweden, and United Kingdom: an expert in the field of regulations and regulatory requirements and the national ECRIN European Correspondent (ECRIN staff trained in clinical research and located in the national coordinating hubs) [27]. For each of the categories of research, the following questions were asked to the Working Group members:

- what is the definition of an investigational product?

- what competent authorities exist in your country?

- is a submission to competent authority required (specify the name of the competent authority and who is responsible for the submission)?

- what are the timelines?

The Working Group members answered the questions by consulting national law documents, web-sites, conducting telephone interviews, etc. The responses to the questionnaire were circulated to the Working Group on regulation and interaction with competent authorities. The results were discussed during several teleconferences and in face-to-face meetings in Paris (19 and 20 May 2007) and Brussels (19 and 20 May, 2008). Moreover, additional teleconferences were held between one of the chairs (JD-M) and national representatives in order to discuss specific national aspects in depth [27]. We also conducted a validation step with the national representatives from the competent authorities of the involved countries.

## Results

### Definition of medicinal product and investigational medicinal product

The Directive 65/65/EEC [29] and the Directive 2001/20/EC [17] defines a medicinal product as: "Any substance or combination of substances presented for treating or preventing disease in human beings or animals. Any substance or combination of substances which may be administered to human beings or animals with a view to making a medical diagnosis or to restoring, correcting or modifying physiological functions in human beings or in animals is likewise considered a medicinal product." The Directive 65/65/EEC has been repealed by the Directive 2001/83/EC [30].

The Directive 2001/20/EC [17] further gives the following definition of the investigational medicinal product: 'a pharmaceutical form of an active substance or placebo being tested, or used as a reference in a clinical trial, including products already with a marketing authorisation, but used or assembled (formulated or packaged) in a way different from the authorised or when used for an unauthorised indication or when used to gain further information about the authorised form'. More specific definition of what constitutes an investigational medicinal product and what constitutes a non-investigational medicinal product has been published by the EU Commission [31].

Our survey collected the definition of an investigational medicinal product used by the competent authority in the ten countries (Table 2). Only Austria adopted the definition directly from the Directive 2001/20/EC. The other countries adopted definitions that were similar but not identical, leading to possible difficulties in multinational trials when a product is considered as an investigational medicinal product in some countries and not in others (for instance, on adverse event reporting, labelling, purchasing of the investigational medicinal product). However, a recent guidance on investigational medicinal products and non-investigational medicinal products was issued in March 2011 to further harmonise the national regulations [32].

**Table 2:** National definitions of an investigational medicinal product.

Countries	Definition of investigational medicinal product
Austria	The definition is that of the Directive 2001/20/EC.

Denmark, France, Hungary, Ireland, Italy, Spain, Sweden, and the UK	The investigational medicinal product is the study drug and the comparator including the placebo or active drug.

Denmark	The rescue drug and all background treatment that directly influences the main efficacy outcomes of the study are also considered investigational medicinal products.

France	The background treatment is also considered an investigational medicinal product if collecting information on it is one of the objectives of the study.

Germany	The investigational medicinal product is a pharmaceutical form of active pharmaceutical substances and placebos, that is tested in a clinical trial on humans or used as a comparator or that is applied to induce specific reactions in humans. This includes EU authorised drugs if they are investigated within a clinical trial, EU authorised drugs if they will be used as comparator, and EU non-authorised drugs.

Italy	The drugs which are not the direct subject of the experimental design, but their use is considered in the protocol, are also considered investigational medicinal products: 1. Drugs with market authorisation, used according to the indications, included in the protocol as needed to the success of the trial, such as drugs to prevent or treat side effects of the investigational medicinal product. 2. Drugs with market authorisation, used outside the approved indication. 3. Drugs without market authorisation, but with market authorisation in other countries of the EC, used within or without the approved indication. 4. Challenge agents, i.e., drugs that are used to induce physiological reactions needed to evaluate the effect of the investigational medicinal product. The rescue drug, and background treatments are not investigational medicinal products.

Spain	Background treatment, the rescue drug, the challenge agent and the medicine used to assess the primary endpoint, if not authorised in any EU country, or when authorised and used for non-authorised indications are also considered investigational medicinal product.

Sweden	The drugs used to assess outcome measures are also considered investigational medicinal products. This includes already approved drugs, which have been formulated differently or are used outside their approved indication, or used to gain additional knowledge about the approved indication.

Submission of clinical trial protocols on typical investigational medicinal products to the competent authority in all ten countries require authorisation for clinical trials using typical investigational medicinal products by competent authorities. However, since April 1^st ^2011 in the UK, the Medicines and Healthcare products Regulatory Agency (MHRA) only requires notification when the risk is equivalent to the risk of usual care-basically trials comparing marketed drugs within the licensed indication [33]. The regulatory authorities, which assess the applications, are presented in Table 3 and can also be accessed via the website of the Clinical Trials Facilitation Group [34]. The sponsor is responsible for the submission of a clinical trial authorisation application as defined by the Directive 2001/20/EC [17]. Any substantial amendment must also be submitted to the competent authority.

**Table 3:** Competent authorities for clinical trials on typical investigational medicinal products in ten EU countries.

Country	Competent authorities for typical investigational medicinal products
Austria	The "Bundesamt für Sicherheit im Gesundheitswesen" (BASG, Federal Office for Health Safety) [42], supported by the Austrian Medicines Agency, AGES PharmMed [43].

Denmark	The Danish Medicines Agency [44].

France	Agence Française de Securité Sanitaire des Produits de Santé (AFSSAPS) [45].

Germany	The Federal Institute for Drugs and Medical Devices (BfArM) [46], and the investigator has also to submit the protocol to the local competent authority.

Hungary	The National Institute of Pharmacy (NIP) [47].

Ireland	The Irish Medicines Board (IMB) [48].

Italy	All clinical trials have to be declared on the database of the Agenzia Italiana del Farmaco (AIFA) (Osservatorio Nazionale Sulla Sperimetazione Clinical Dei Medicinali; National Monitoring Centre for Clinical Trials) [49]. For phase I clinical trials, the competent authority is the Istituto Superiore della Sanita (ISS) [50]. For phase II, III and IV clinical trials, the competent authority is the director of the Public Health Facility [51].

Spain	The Spanish Agency for Medicines and Medical Devices (AEMPS) [52]. Performance of clinical trial on medicinal products not authorised in the EU and containing any active substance not included in any authorised medicinal product in Spain requires an additional application for a 'product under clinical research qualification' (PEI).

Sweden	The Medical Products Agency (MPA) [53].

The UK	The Medicines and Healthcare Products Regulatory Agency (MHRA) [54].

The deadlines for accepting or rejecting a clinical trial application by the competent authority are 30 days from receipt of valid application to accept, accept with conditions, or reject. The final decision is taken within 60 days of the original request. Only one cycle of correspondence on any queries, which arise from the assessment, is allowed. If no answer is received from the sponsor or if the answer is not acceptable, the application is refused. Opportunities exist for dialogue with the national regulatory authorities to ensure the tasks of the sponsor and questions to the sponsor are adequately addressed. Divergences, however, still exist in the documentation and national requirements, and in the decisions of the national competent authorities. For this reason, the heads of medicines agencies implemented in 2010 the Voluntary Harmonisation Procedure, offering the possibility to apply through a single dossier and to undergo coordinated review for multinational trials [35].

## Discussion

Our results show that clinical trials using typical investigational medicinal products require authorisation from a competent authority in each of the ten EU countries surveyed. The opinion of the competent authorities is communicated to the trial sponsor within the same timelines in all ten countries surveyed. This is a clear indication that the requirements of the Directive 2001/20/EC [17] have been relatively uniformly adopted for this type of clinical research in these ten countries, and their application structure, that is the institutions one has to apply to, is comparable but not identical. The overall result of the implementation of the Directive 2001/20/EC is harmonisation of requirements for typical investigational medicinal products and a navigable landscape for the applicant.

In practice, this harmonisation of regulatory requirements is in fact specific to a certain type of investigational medicinal product, which we name 'typical investigational medicinal product'. In practice, the regulatory requirements are not harmonised for a number of other important investigational medicinal products, which we name 'less typical investigational medicinal products' [27], including different sub-categories of medicinal product as listed and described in the Directive 2003/63/EC [36]:

○ Biological medicinal products, i.e., proteins preceded either from biological material by standardised purification techniques or by recombinant DNA technologies or hybridoma or monoclonal antibody methods.

○ Vaccines.

○ Advanced therapy medicinal products, consisting of

- gene therapy medicinal products (human or xenogeneic);

- somatic cell therapy medicinal products (human or xenogeneic);

- tissue engineered product, i.e., a product that contains or consists of engineered cells or tissues (human or xenogeneic).

○ Plasma-derived medicinal products.

○ Blood products.

○ Radio-pharmaceuticals and precursors.

○ Homeopathic medicinal products.

○ Herbal medicinal products.

The seemingly uniform definition of an investigational medicinal product from the Directive 2001/20/EC [17] did not prevent many sub-categories of medicinal products to be selected out across the EU. This gave rise to specific regulatory requirements across EU Members States. These specific regulatory requirements have increased the complexity of the national clinical trial regulation in the EU [28] and participated in the creation of a chaotic regulatory environment which threatens ethical and fairness considerations both to patients and researchers [15,16,18-26]. The chaotic regulatory environment, in which intervention decides regulation [28], alienate researchers from understanding the basis for implementing interventions into clinical practice and this puts patients at risk.

The definition of a 'medicinal product' in the Directive 65/65/EEC [29] and reiterated in the Directive 2001/20/EC [17] and the Directive 2001/83/EC [30] is much clearer, comprehensive, and fairer than the definition of an 'investigational medicinal product' in the Directive 2001/20/EC [17]. The former Directives defines a medicinal product as: "a) Any substance or combination of substances presented as having properties for treating or preventing disease in human beings; or b) Any substance or combination of substances which may be used in or administered to human beings either with a view to restoring, correcting or modifying physiological functions by exerting a pharmacological, immunological or metabolic action, or to making a medical diagnosis." However, the Directive 2001/20/EC [17] defines an investigational medicinal product as: "a pharmaceutical form of an active substance or placebo being tested or used as a reference in a clinical trial, including products already with a marketing authorisation but used or assembled (formulated or packaged) in a way different from the authorised form, or when used for an unauthorised indication, or when used to gain further information about the authorised form".

We would very much have preferred that the definition of an 'investigational medicinal product' built upon the wording for the definition of a medicinal product used in the Directive 65/65/EEC [29], Directive 2001/20/EC [17], and Directive 2001/83/EC [30], e.g.,: a medicinal product (as defined by the three cited Directives) with potential beneficial properties being tested, or used as a comparator reference, in a clinical trial. Including products already with a marketing authorisation but used or assembled (formulated or packaged) in a way different from the authorised form, or when used for an unauthorised indication, or when used to gain further information about the authorised form.

There is a huge amount of legislation and guidance pertinent to clinical research in the EU as well as in the different member states; this makes it difficult to have an overall view of the full regulatory requirements [17,30,37-39]. An overview of the kind presented here is novel and provides a necessary overview of the regulatory framework within the ten EU countries participating in ECRIN during the 2006 to 2008 period regarding typical investigational medicinal products. To our knowledge this is the first time that a survey about the regulatory basis for typical investigational medicinal products has been conducted in such depth.

The present study has a number of weaknesses. First, we only assessed ten countries. Although we covered the majority of the EU population (70%) there are still 17 EU countries not considered. ECRIN is in the process of linking to the national clinical trial networks in these countries and ECRIN will assess their regulatory environment regarding typical investigational medicinal products [30]. Second, we only assessed the regulatory authority structure during the approval phase of the trial in the surveyed countries, not how the regulatory process was working during the conduct and termination of the trial. Third, we did not go into details about how other aspects of the Directive 2001/20/EC, e.g., good clinical practice were implemented and functioned. Fourth, we only concentrated on how the typical investigational medicinal product was handled in the national laws and regulations. The latter also explains why the present study observes relative harmonisation compared to other assessments of the implementation of the Directive 2001/20/EC, which found divergences at the national level and increased complexity in conducting multinational clinical trials [18-26]. In our assessments of less typical investigational medicinal products we observed substantial heterogeneity [28].

Compared to typical investigational medicinal products, the regulatory landscape is not so navigable for other categories of clinical research [27,28]. Although, strictly speaking, all investigational medicinal products are legislated for under the 2001/20/EC Directive and the definitions therein, less typical investigational medicinal products such as biopharmaceutical investigational medicinal products or other less typical investigational medicinal products investigating gene therapy, tissue engineering, cell therapy, blood-derived products, monoclonal antibodies, recombinant proteins, vaccines trials, etc [27] all necessitate additional regulatory requirements, which vary across Europe [28]. In short, the regulatory environment is much more heterogeneous and thereby problematic for academic trialists and small and medium sized companies regarding other medical interventions [28]. This puts at stake the interests of patients [25,26].

Regulatory requirements should be in place with the principal objective of protecting the participants and ensuring their safety, and that regulatory requirements can be adjusted according to the risk of the trial [28]. However, it appears that regulatory requirements in different Member States are driven trivially by the category of the intervention and not on the overall risk of the trial [27,28]. An innovative biopharmaceutical product could be equally as high risk as an innovative surgery. Risk is a complex and multi-faceted concept and can be influenced by, among other things, vested interests (sponsorship), the size of the trial (feasibility), the number of centres (feasibility and quality assurance), and crucially, and the nature of the experimental and control interventions [40]. Therefore, a risk based approach rather than an approach depending on type of investigational medicinal product seems a better way to determine the need for security and monitoring in a given clinical trial [24,41]. Interestingly a pilot initiative steered by the MHRA, the MRC, and the Department of Health started in April 2011 the UK, defining risk assessment and risk-adapted supervision in the framework of the Directive 2001/20/EC [33].

The Voluntary Harmonisation Procedure implemented since 2010 represents a substantial progress for the regulatory supervision, allowing coordinated review for multinational trials [34]. We ought to go further and implement a single application and authorisation process for all clinical trials, by which the sponsor submits one electronic application which is accessible by the competent authorities, the ethics committees, and the public, with harmonised definitions of the roles of the ethics committees (protection of the trial participants) and of the competent authorities (assessment of the health product) [28,35].

## Conclusions

The Directive 2001/20/EC seems to have delivered on its harmonisation objective regarding typical investigational medicinal products during the application phase; we commend this achievement. However, it only benefits sponsors and investigators conducting clinical trials on typical investigational medicinal products. We cannot have a hierarchical system where the regulatory requirements are relatively harmonised and navigable for one set of clinical interventions and a minefield of country-specific barriers for other clinical interventions [28]. Our results show that the harmonisation is limited to the field of typical investigational medicinal products [28]; this harmonisation does not even encompass all investigational medicinal products that Directive 2001/20/EC itself defined. Only a European regulation, binding in their entirety to and immediately applicable in all EU member states, can truly harmonize the rules for clinical research in Europe. De-fragmentation of the authorisation procedure as well as risk-based clinical trial supervision and monitoring processes should be key elements in such new legislation.

## Competing interests

None of the authors have financial or non-financial competing interests.

## Authors' contributions

CK, JDM, and CG drafted the survey and conducted the analyses of the results. All the authors participated in the development of the survey and discussion of the results. JDM, CK, KW, and CG drafted the manuscript. All the authors have read and corrected draft versions of the manuscript and approved the final version.
